# Addressing victimization to enable societal participation in flexible assertive community treatment: A process evaluation of the implementation of a new intervention

**DOI:** 10.3389/fpsyt.2022.956133

**Published:** 2022-09-20

**Authors:** Wendy M. M. Albers, Yolanda A. M. Nijssen, Diana P. K. Roeg, Jaap van Weeghel, Inge M. B. Bongers

**Affiliations:** ^1^Tranzo, Scientific Center for Care and Wellbeing, School of Social and Behavioral Sciences, Tilburg University, Tilburg, Netherlands; ^2^Kwintes Supported Housing, Zeist, Netherlands; ^3^Parnassia Group Academy, Parnassia Psychiatric Institute, The Hague, Netherlands; ^4^Phrenos Center of Expertise for Severe Mental Illness, Utrecht, Netherlands; ^5^Research Group Evidence Based Management of Innovation, GGzE, Mental Health Care Institute Eindhoven, Eindhoven, Netherlands

**Keywords:** victimization, participation, stigma and discrimination, severe mental illness, mixed-methods design, process evaluation, rehabilitation, recovery-oriented care

## Abstract

**Background:**

Individuals with severe mental illness experience more victimization and discrimination than other persons in the community. Effective rehabilitation and recovery-oriented care interventions aimed at addressing this issue are lacking. We therefore developed a victimization-informed intervention (accompanied by a training module for professionals) called *the Victoria intervention*. The purpose of the present study was to understand the trial effects by examining the implementation process and the factors that influenced it.

**Materials and methods:**

A process evaluation was conducted using a mixed-methods design. During the professionals’ intervision sessions, we used observations to understand the learning processes (*n* = 25). Subsequently, we studied the use of the intervention in practice through structured questionnaires (*n* = 215) and semi-structured interviews (*n* = 34) with clients and professionals. We used descriptive and inferential statistics for the quantitative data and the framework method for the analyses of the qualitative data.

**Results:**

The observations showed that the trainings were well received. The professionals shared the urgency of paying attention to victimization and discrimination and its harmful effects on participation. They also found the intervention steps to be logical and the intervention protocol easy to use. Nevertheless, they mentioned in the interviews that they had experienced difficulties initiating a conversation about victimization, and if they started one, they did not always follow the steps of the intervention as intended. Few clients said that victimization was placed on the agenda, though those who had discussed victimization with their caregivers expressed their appreciation in the interviews; they felt acknowledged and supported.

**Discussion:**

The findings indicate that the intervention was considered helpful in raising awareness and the acknowledgment of victimization. However, professionals remain reluctant to talk about the subject, and the results show they need more practical training in this regard. This process evaluation has an important added value in that it helps us to understand the results of the effect evaluation of the intervention. The findings will facilitate the development and implementation of interventions that address clients’ victimization experiences in community mental healthcare settings and subsequently enable their participation in society.

## Introduction

Individuals with severe mental illness (SMI) are victims of crime and discrimination more often than other citizens (1–5). The consequences of victimization, discrimination, and stigmatization can worsen symptoms and additional mental health issues, re-victimization, perpetration, and social isolation (1, 6, 7), which are considered important barriers to societal participation and recovery (6, 8, 9). While evidence exists that understanding and addressing why a client is demoralized by previous victimization experiences are beneficial for rehabilitation and recovery (10–14), effective tools to recognize and address victimization experiences, including (anticipated) stigmatization and discrimination, are lacking. We developed and tested the Victoria intervention, a four-step intervention that aims to increase safe societal participation by increasing awareness and the acknowledgment of victimization (15). The effect study was published in a previous article (16).

Several characteristics of the Victoria intervention, the trial, and the results made it important to carry out a process evaluation. First, we wanted to understand the trial effects as we performed a cluster randomized controlled trial (RCT) with the intervention in flexible assertive community treatment (FACT) teams (17, 18). Although we found that the intervention successfully moderated experienced discrimination, it had no effects on participation and victimization or on other primary outcome measures, contrary to our expectations (16). Nevertheless, clients felt that their victimization experiences were acknowledged and that they were supported in the recovery process. In both the intervention and control groups, anticipated discrimination and self-efficacy increased slightly over time. However, even though previous studies have reported that participants feared relapse after discussing their victimization experiences (19, 20), psychosocial functioning, victimization, and other outcomes neither worsened nor increased in the intervention group. Conducting a process evaluation was warranted to help understand these mixed findings and move forward with the intervention in practice (21, 22).

Second, the design of the Victoria intervention makes it difficult to measure its use in daily practice. It trains professionals to discuss victimization experiences with their clients, and we expect clients to feel acknowledged and supported in their recovery process (i.e., for the intervention to have an indirect effect). What happens in the process is not easy to quantify in an effect evaluation. The degree of flexibility that is built into the intervention adds to this complexity. By executing a process evaluation, we can obtain knowledge about the extent to which professionals recognize and acknowledge victimization in their clients (22, 23).

Third, practical trials, such as the one in which the Victoria intervention was tested, are characterized by multiple interacting components, and the context, which may partially determine the outcome is difficult to control for (24). For example, we trained the professionals in the FACT teams and we measured the effects for 20 months. In this timeframe, we could not rule out any change of personnel or conversations between teams. Furthermore, the trial was performed in multiple sites, each of which had specific contexts that may have influenced outcomes (25). In these complex settings, whether the intervention works is naturally important, but contextual influences on implementation or outcomes also have to be examined to gain deeper insights (21, 25). Gathering the perspectives of all stakeholders can provide knowledge of these contextual effects. Moreover, process evaluations can help us understand why certain effects are obtained by measuring the response of the intervention among clients and professionals (23, 25).

Hence, by examining the implementation process (including training and use), the factors that influenced it, and the impact the intervention had on the clients, we aimed to understand the trial effects.

## Materials and methods

### Design

For this process evaluation, which was embedded in a previously published cluster RCT (26), we used a mixed-method design using both qualitative and quantitative data (see Table 1 for the research questions and accompanying data sources). First, as the intervention was interwoven in the rehabilitation work of the FACT team, treatment plans for clients from both the intervention and control groups were used to measure adherence to rehabilitation principles in general. Second, the intervision sessions (i.e., meetings coached by a trainer to discuss the conversations they have had in their daily work) following the training were audio-recorded so we could assess the extent to which the professionals used the intervention in daily practice, how and when they used it, and the factors that influenced its use. Third, for the quantitative part of this study, data from structured questionnaires among clients and professionals were analyzed to examine perceptions of clients’ conversations with their case managers and whether the professionals concerned were aware of their clients’ victimization experiences. Fourth, the professionals were asked to fill out a checklist during the intervision sessions to see how faithful they had been to the intervention itself. The checklist was designed in the development phase of the intervention. Finally, semi-structured interviews were conducted with professionals of the intervention teams, management, and trainers to gather information about their experiences of the training sessions, influences on the use of the intervention, how and when they used the intervention, and the impact of the intervention on the client from the perspective of the professional. Semi-structured interviews were held with clients to cross-check the use and impact of the intervention. The study protocol of the RCT was approved by the Medical Ethical Committee of the Elisabeth Hospital in Tilburg (NL53845.028.15) on November 18, 2015 for all participating sites. The study was registered with the Dutch Trial Register (NTR 5585).

**TABLE 1:** Research questions and relevant data sources.

		Treatment plans	Observations of intervision meetings	Structured questionnaires (professionals)	Structured questionnaires clients	Checklist (professionals)	Interviews with professionals	Interviews with clients
Rehabilitation principles	To what extent do professionals work according to rehabilitation principles?	x						
Training	What were the experiences with training and intervision meetings?						x	
Use	To what extent did the professionals use the intervention?		x	x		x	x	x
	How did they apply the intervention?		x				x	
	When did they apply the intervention?		x				x	
	What factors influenced the use of the intervention?		x				x	
	What was the perceived impact of the intervention on clients?		x		x		x	x

### The intervention

The Victoria intervention was designed to be victimization informed and to serve as an add-on module so that professionals can be made aware of and sensitive to the topic while employing rehabilitation approaches. It supports professionals in starting and structuring conversations with their clients regarding their experience of victimization and how it impacts their societal participation. The intervention comprises four steps: exploration, analysis, clarifying the context, and future strategies (15). The first step involves exploring the client’s societal participation and their satisfaction with it. When a client is avoiding societal participation or if their desired progress is stagnating, continuing to the second step is indicated. During the second step, relevant negative experiences related to societal participation are discussed. A narrative approach is adopted in which the professional acknowledges the pain of the client’s victimization experiences (27) and tries to understand the avoidance or stagnation. The third step is to clarify the context of the experience, that is, why the client initially engaged with the situation in which they were victimized. The objective of this step is to switch from the negative experience to a more positive perspective as taking reasonable risks is necessary to progress in life. The fourth step is to conclude the conversation by determining future steps. This can vary from planning another conversation, reformulating a rehabilitation action plan, starting other interventions such as supported employment (e.g., IPS) or treatment for self-stigma (e.g., NECT), or, when there is underlying trauma, trauma-focused therapy.

Training consisted of a refresher on the rehabilitation method that had been used previously. This was followed by three sessions on the Victoria intervention for the entire team. The first session comprised securing the intervention on an individual and team level. The second and third sessions were provided as per the respective organizations. In the first session, ways were discussed to implement and secure the intervention as part of the participants’ daily routine. The second and third sessions comprised an explanation of the steps and role-playing practice. Finally, intervision meetings were held every 6–8 weeks. During these, the conversations the professionals had been having with their clients were discussed (with the assistance of a trainer). A short handout was designed to be used during conversations with clients.

### Participants

The participants comprised mental health nurses, experts-by-experience, managers, and clients of eight FACT teams. The trainers were also invited to take part in qualitative interviews. For an overview of all participants and other data sources, see Table 2.

**TABLE 2:** Data sources included in the analyses.

Data source	*N*	Average duration^a^
Treatment plans	66	–
Observations		
Intervision meetings	25	50
Structured questionnaires		
Clients and professionals	215	–
Checklists	20	–
Qualitative interviews (*n* = 34)		
Clients	16	28
Mental health nurses	7	39
Experts-by-experience	5	39
Managers (of which one was also a psychiatrist)	3	37
Trainers	3	48

^a^Time is in minutes.

Table 3 lists the characteristics of the study participants in both the subsample for the qualitative interviews, as well as the full sample from the structured interviews from the RCT. In the subsample, half of the client participants were males, and the average age of the clients was 45. Most of the clients had a psychotic or mood disorder as a primary diagnosis. In the full sample, most had schizophrenia; 56.2% of the clients had been the victims of one or more incidents of crime in the previous year, and 46.1% of the clients in the RCT sample. Ten of the professionals were male and eight, female.

**TABLE 3:** Characteristics of the interviewees at T2.

Clients	Subsample (*N* = 16)	Full sample (*N* = 332)
**Gender**		
Male	8 (50%)	199 (59.9%)
Female	8 (50%)	133 (40.1%)
**Age at beginning of the study**		
<30	–	12 (3.6%)
30–39	5 (31.3%)	63 (19%)
40–49	5 (31.3%)	117 (35.2%)
50–59	6 (37.5%)	106 (31.9%)
>60	–	34 (10.2%)
**Education**		
Low	7 (43.8%)	160 (48.8%)
Middle	5 (31.3%)	125 (38.1%)
High	2 (12.5%)	43 (13.1%)
**Living situation**		
Living at parents	–	7 (2.1%)
Supported housing	–	38 (11.6%)
Living on their own	16 (100%)	283 (86%)
Other	–	1 (0.3%)
**Born in the Netherlands**		
Yes	13 (81.3%)	287 (86.4%)
No	3 (18.8%)	45 (13.6%)
**Marital status**		
Not married	8 (50%)	218 (65.9%)
Divorced	4 (25%)	58 (17.5%)
Married	4 (25%)	45 (13.6%)
Widow/widower	–	8 (2.4%)
Living together	–	2 (0.6%)
**Employment status**		
Benefits	15 (93.8%)	249 (75%)
State pension	–	3 (0.9%)
Employed	1 (6.3%)	62 (18.7%)
Other	–	18 (5.4%)
**Primary diagnosis on T0**		
Schizophrenia	1 (6.3%)	114 (28.4%)
Other psychotic disorder	6 (37.5%)	99 (24.7%)
Mood disorder	4 (25%)	49 (12.2%)
Anxiety disorder	1 (6.3%)	27 (6.7%)
Developmental disorder	1 (6.3%)	38 (9.5%)
Substance use disorder	–	5 (1.2%)
Other diagnosis on AxI	1 (6.3%)	13 (3.2%)
Personality disorder	2 (12.5%)	56 (14%)
**Professionals (*N* = 18)**		
**Role**		
Mental health nurses (case manager)	7 (38.9%)	–
Experts-by-experience	5 (27.8%)	–
Managers (of which one is also a psychiatrist)	3 (16.7%)	–
Trainers	3 (16.7%)	–
**Gender**		
Male	10 (55.6%)	–
Female	8 (44.4%)	–

### Procedure

All qualitative interviews were conducted between April and July 2018, during the 20-month follow-up period of the RCT. For the professionals, a purposive sample per team of three mental health professionals, including one expert-by-experience, was recruited to reflect a diverse range of opinions on the intervention. As the number of trainers and managers was limited, all were asked to participate, and all agreed.

From the clients who participated in the RCT and were exposed to the intervention at the request of their case manager, a random sample of 24 was contacted via telephone for a qualitative interview, and 16 agreed to participate. We based the number of clients needed to contact on several factors, such as the research questions, the nature of the interview topics (questions were rather specific), the use of other data (i.e., triangulation), and practicality (28, 29). Additionally, by taking into account a 15% attrition rate, we invited one extra client per intervention team. Non-participation had no consequences in terms of the care they received. The interviews were held at a location of the participant’s choosing (i.e., either home or FACT team office). Before the interviews took place, written consent was requested. The interviews with both the professionals and clients were led by an interview guide. Topics were based on the intervention components (15) and a framework for process evaluations for cluster RCTs (25). Since we wanted to elicit specific information from each of the groups, different interview guides were developed for each group. The guide for the professionals included their perspectives on the intervention, their ideas regarding its usability and degree of usage, and their ideas on clients’ experience of the intervention. For the clients, the guide included their experience of conversations (with their case manager) and their difficulties with societal participation and victimization.

To ensure their objectivity, the interviews were carried out by two research assistants who were not involved in the development of or training in the intervention. They had training in interviewing and discussed issues that emerged after the first interviews. The interviews were audio-recorded unless otherwise requested by the participant, and were pseudonymized. On average, the interviews with the professionals lasted 40 min, and with the clients, 28 min (see Table 1). The clients received financial remuneration of €10.

The structured questionnaires were collected during the effect evaluation within the RCT at T1 (10-month follow-up) and T2 (20-month follow-up). The professionals’ questions addressed the extent to which they had used the intervention in daily practice with each of the clients in the study and the insight gained into how victimization acted as a barrier to clients’ societal participation. The clients’ questions addressed the extent to which they felt they were being listened to and supported in their recovery process.

The intervention teams had on average 7–9 intervision meetings. Eighty percent (25 out of 31) of the intervision meetings were audio recorded (six were missing, for practical reasons unrelated to the study questions). A trainer, professionals from the intervention team, and a researcher participated in each meeting.

Pseudonymized treatment plans from a random sample of 15% of the clients participating in the RCT were collected at T0 (baseline) and T2, resulting in 63 treatment plans at T0 and 40 plans at T2. Adherence to rehabilitation principles was measured by the method developed by Wunderink et al. (30). This involved scoring the overall treatment plan (plan level) according to five criteria: whether goals were formulated; whether the treatment plan was written in the first person; whether there was space for clients’ consent; whether evaluation dates had been planned; and whether emergency agreements were available. Furthermore, the treatment goals (goal level) of the following nine life domains were scored: housing; work and occupational activities; education; recreation; social contacts; meaning in life; self-care; mental health; and physical health. Each goal area is scored according the choose-get-keep model of psychiatric rehabilitation (31): is the rehabilitation phase clear; is there a specified timeline; is there a clear task division; and is the client’s role clear. Finally, the overall quality score of the plan was calculated by adding up the items on plan level and the goal level.

Finally, the professionals in each team were asked to fill out a fidelity checklist including the objectives of each step of the intervention by using a four-point scale ranging from *not at all* to *completely* to measure the extent to which they followed each step and whether they met the accompanying objectives.

### Analyses

All interviews and observations were audio-recorded and transcribed verbatim. Analyses were performed using the framework method (32), a form of thematic analysis consisting of five distinct phases: familiarization; identifying a thematic framework; coding and indexing; charting; mapping; and interpretation (33). The method is particularly suited for comparing and contrasting qualitative data, and due to the distinct steps, analyses are transparent and reproducible (33).

Atlas.ti was used for the initial open coding of a sample of the interviews, which were coded by the first author and discussed afterward with the second and third authors to ensure that all agreed with the codes. Categories and over-arching themes were identified; they formed the analytical framework on which the rest of the interviews were coded by the first author. Themes and subthemes were summarized in the form of charts and a framework matrix for interpretation and comparison. These were discussed with all authors. The results are reported according to the COREQ checklist (34). Data from the structured questionnaires for the clients and professionals in the intervention and control groups, the treatment plans, and the checklist were analyzed using descriptive statistics. All quantitative analyses were performed using SPSS Version 24.

## Results

The adherence to rehabilitation principles is shown for the 125 clients in both the control and intervention condition at baseline and 20-month follow-up (Supplementary Table 1). In almost all treatment plans, one or more rehabilitation goals were formulated. The prevalent treatment goals were (from most to least mentioned) mental health; self-care; social contacts; work; meaning in life; physical health; housing; daytime activities; and learning. The overall quality of the treatment plans was 5.2 (on a scale from 1 to 10). Most of the plans revealed insufficient adherence to rehabilitation principles; in no case was there substantial or full adherence.

In the following sections, the major themes regarding the implementation process and its influencing factors identified in the interviews with professionals, managers, and trainers are discussed, together with additional information from the intervision meeting observations and the structured questionnaires. The themes were: (1) attitudes toward and reasons for discussing victimization; (2) the process of implementation; (3) factors affecting the use of the intervention; and (4) the perceived added value of the intervention. The results from the client interviews are also presented.

### Attitudes toward and reasons for discussing victimization

Victimization was considered by all the participants to be an important issue for discussion. The professionals described three reasons for using the Victoria intervention. First, victimization is seen as something inherent in people with severe mental health problems, and therefore the topic should be embedded in one’s daily routine. Several of them argued that all of their clients experienced victimization on a regular basis, with some stating that victimization will become a greater problem in the future because of the changing care system and growing intolerance toward people with mental illness. One professional said that

Those people all have a history. A lot of have experiences and you’re not here for no reason. And um… so the chances are, if you’re at FACT and you have victimization experiences, those chances are pretty high (P15).

A second reason was that the intervention focuses on an undiscussed or oft-avoided topic. Victimization is not an easy subject to discuss and is sometimes avoided by both clients and professionals. One professional suggested that because victimization is inherent to the FACT population, many relate it to their psychopathology. Another professional confirmed that the topic often remains undiscussed: “In three-quarters of an hour you have to look at so many things. I notice a very conscious reflection on these things, that I think this happens far too little” (P12).

Yet another reason was that, according to the professionals, victimization has an enormous impact on participation. They recognized that clients sometimes refrained from undertaking activities because they want to avoid disappointment. Many clients “have become so familiar with disappointments that it is more or less an expectation that things would turn out that way” (P4). Several argued that supporting social recovery is what the job of a case manager is all about, so victimization should be given more priority.

However, a few professionals had some doubts about the intervention. One argued that due to high workloads and a lack of staff, the priority should be on treatment and not on barriers to rehabilitation in cases of addiction and personality disorders. Another expressed her concerns about focusing on victimization thus:

But with the emphasis you put on victimization um… you might also increase the likelihood that it’s precisely about that. You know what I mean, right? So people sometimes don’t move because they are victimized and so we have to pay attention to that. And then it’s a benefit. Um… but because you emphasize it so much, it can also be that… and that’s a bit… you know, social workers… social workers love trauma and victimization, huh? (P6).

#### Comparison with care as usual

All experts-by-experience indicated that the steps of the Victoria intervention were nothing new. They already focused on victimization in their conversations with clients, asking about previous (negative) experiences and what preceded them.

But I have a, an easier entrance, I think, because I get to talk about my own experiences as well… I’ve been through um a lot of traumas. And also psychoses from traumas. So yes, then…it’s already a bit natural that I’m really curious about how they…what they’ve been through, how their eh life situation is, eh…what they need, what they eh, eh…yes, I investigate that with the clients completely (P1).

Most of the case managers admitted they addressed victimization with their clients, but not on a regular basis or as extensively as the Victoria intervention. A commonly expressed view was “I did have these kinds of conversations, but never so precise.” One admitted the following:

I must say that I uhh, that I do find it a… in that sense a useful or a beautiful intervention, because it (victimization) uhh maybe by some people often underexposed, while I myself always uhh without knowing the intervention already had the idea that I focused on people’s recovery and why they do not come to recovery so to speak (P14).

Finally, the case managers all acknowledged that they often wanted change too fast too soon, or that they often started offering solutions.

### Process of implementation

#### Extent of application

Results from the structured questionnaires (Supplementary Table 2) show that, according to the professionals, 55% and 60% of clients were exposed to the intervention at T1 and T2, respectively. The professionals stated that most of their clients had the occasional Victoria conversation (52% at T1 and 48% at T2). A small percentage had a Victoria conversation often (15% at T1 and 11% at T2). The extent to which professionals discussed Victoria conversations with colleagues increased over time, from 29% at T1 to 43% at T2.

The professionals reported in the fidelity checklist that they generally went through all the steps of the intervention, with a sum fidelity score of 3.06 (with scores ranging from 0 to 4; Table 4). The two lowest-scoring items were responses to “To what extent are you not going along with the avoidance?” and “To what extent can you say for yourself that you have not been too quick to think in terms of solutions?” This suggested that they had the most difficulty applying these components of the intervention.

**TABLE 4:** Checklist of the steps of the intervention.

To what extent… Mean score (SD)^a^	Mean score
… do you know how this client is doing in terms of living, social contacts, and activities?	3.16 (0.96)
… do you have any insight into whether there is any avoidance of or stagnation in (social) activities?	3.21 (0.71)
… do you have insight into whether or not victimization experiences or other kinds of setbacks are linked to participation?	3.21 (0.71)
… are you not going along with the avoidance?	2.11 (0.57)
… do you have a clear picture of the experience?	3.11 (0.81)
… can you understand the intensity of the client’s feelings?	3.21 (0.79)
… do you understand the causes (in terms of client behavior, the behavior of others, and the circumstances)?	2.95 (0.78)
… did you give the client sufficient recognition and understanding of the (causes of) their experience?	3.16 (0.96)
… do you understand discouragement and/or avoidance?	3.37 (0.83)
… do you have an idea of the client’s desires that lay beneath their experience?	3.32 (0.48)
… do you have an idea of what the client was hoping to achieve?	3.16 (0.96)
… have you discussed whether another conversation about this experience is desirable?	3.37 (0.90)
… have you discussed whether and how to proceed with the original goal?	3.06 (0.87)
… do you have a view on how to proceed?	3.05 (1.03)
… can you say for yourself that you have not been very quick to think of solutions?	2.53 (1.07)
Overall score^b^	3.06 (0.42)
Was it possible to conduct a Victoria conversation with this client? (Yes) *n* (%)	16 (84.2%)

^*a*^Scores ranging from 0 (*not at all*) to 4 (*completely*). ^*b*^Minimum = 2.20, maximum = 3.53.

By contrast, in the qualitative interviews, the professionals indicated that they did not use the intervention extensively in their daily practice. While they valued it and thought that more attention should be paid to barriers to participation, they were unable to use the intervention as much as they would have liked to. The same can be stated for the teams. Most of the professionals explained that in their teams, neither the intervention nor victimization were discussed on a regular basis. Only in one team did clients who had meetings about their treatment plans the following week discuss the question of social functioning. The case manager of that team added that in other team meetings they also asked questions such as “Why is it that this client has issues with participating, why does he not go outside, or why did he quit his education” (P10) more often. According to the professionals, the main reason for not using the intervention in daily practice was because it was new and they were creatures of habit; they were only reminded of it in intervision meetings, after which it faded into the background again. Both trainers and case managers stated that intervision meetings consisted mainly of discussing potential clients rather than evaluating previous Victoria conversations.

#### Steps of the intervention in daily practice

The chief indicator of the Victoria intervention according to the protocol is when a client has issues with societal participation. According to the professionals, 34.4% of the clients were avoiding participation, and 33.6% stopped participating. The professionals confirmed that this was due to victimization in two-thirds of those cases (64% at T1 and 56% at T2; see Supplementary Table 2).

In the qualitative interviews, most of the professionals claimed that they applied the intervention flexibly, for example by starting to address victimization rather than the clients’ struggles with societal participation. Those who used the intervention more thoroughly noticed that it helped to address victimization in terms of participatory stagnation; it opened doors, and they also learned “new” things about their clients: “… while if you go more in the direction of, “Yes, but it’s terrible what happened to you,” that is easier for them to bear. Then it becomes an easier subject” (P16).

Most experts-by-experience indicated that they used the steps of the intervention as intended, and thought it was *doable* if you take your time. They also said they took a very flexible approach to the sequencing of the steps, as the outcomes of each were related to personal experiences and stories, so required a personalized approach.

They also pointed out that they often recognized certain feelings themselves (e.g., shame and discouragement), and this made it easier for them to relate to the feelings of the client; they therefore understood the importance of the second step in particular. In most case manager interviews, this step was not discussed, though two of them concluded that “What I’ve noticed in particular is that, when I’m working with Victoria … is that, when you ask about this (victimization), I’ve noticed that people are often open about it, if you really pay attention, if you really try to listen” (P10).

Most professionals argued that participation-related victimization was not a topic clients put on the agenda; indeed, they avoided it altogether. A potential issue was that the professionals might along with them. They recognized that they needed to be aware of this and take the initiative to make it a topic of conversation.

The professionals struggled with applying the intervention in daily practice partly because each step required a considerable amount of time. One of the trainers argued that most case managers had a tendency to focus on recognizing and acknowledging negative experiences and offering solutions, to the point where the conversation was no longer about social functioning.

The case managers argued that the tendency to offer solutions too soon was an issue. The necessary time had to be taken to complete the first two steps (i.e., exploration and analysis). One professional stated that “I had to sit on my hands quite often (P10).” This tendency was confirmed in the intervision meetings when cases were being discussed: “I wouldn’t come up with solutions myself, but I would ask “what have you tried?” … then you want to follow through on that as well” (W23).

Finally, beginning the intervention could also be a challenge. Many of the professionals started with a clear suspicion that the client had been victimized. Only a few case managers indicated that they introduced the intervention when a client was struggling with societal participation. They were also the ones who used the intervention quite often. Some experienced difficulties in choosing the right moment; a client had to be in the *right* recovery phase. This was confirmed in the intervision meetings.

#### Usability

In general, the intervention was perceived as clear, logical, and comprehensive. The professionals argued that the steps and the accompanying handouts were easy to use. However, the problem with using the intervention in daily practice lay in a certain reluctance stemming from a fear of inflicting more trauma. They felt that discussing victimization experiences could cause their clients distress, even though most of them agreed that in the long run, this would be beneficial. The case manager of a client who worked as a sex worker on the streets and was suffering from addiction said:

I assume that he has experienced a lot of traumas. But I… also one of those ladies once said to me: yes, I use because I experience so much on the streets. I use to cope. And so I can forget those bad experiences. When I ask someone in depth, Where was it? And how was it? I find that uhm, yes, this does not quite fit my role then. What if I’m rooting around in something that has bothered people for years? You have to know what you’re doing (P11).

Nevertheless, all the professionals said that they referred to a psychologist in their team if they suspected underlying trauma. They were also aware that this, in addition to using the trauma screener, is advised in the fourth step of the intervention. They argued that the intervention is not suitable for everyone. They agreed that for clients with psychosis or heavy addictions, the intervention should wait. Some were more hesitant than others: “If someone drinks 12–13 cans of beer a day, I don’t have to have this conversation. … Then I agree (with the client), if I come by uh, it is in the morning and (we agree) no alcohol” (P9).

Several preconditions for the application of the intervention emerged from the interviews. The most important one was that a narrative attitude requires time. Sitting down with the client and not being led by the delusion of the day creates a space for the client to be open. The downside of this is that professionals have to slow down and take more time with a client than is scheduled. Another precondition is having a connection or a good relationship with the client. This also creates a safe space in which they feel safe to open up about their victimization experiences, though having these kinds of conversations with new clients was not considered advisable.

#### Training

Attendance at the first training session was 97%, followed by 66% and 58% for the second and third sessions, respectively. In general, professionals perceived the training as “well put together.” On the role play and fictitious cases, they were divided. Some thought this was insightful and others found it difficult to practice the intervention in a created situation. Finally, they preferred to have the second and third part of the training per team, instead of per organization.

The intervision meetings were not a priority in most of the teams, which led to a lower attendance rate or even rescheduling meetings. Case managers were too busy and, for example, clients’ crises were of higher priority. This led to lesser continuity across meetings, because cases brought in at one meeting could not be followed up in the next. The input of cases was appreciated though and was perceived as practical and enlightening. However, it remained mostly talking about rather than evaluating actual conversations.

### Factors affecting the use of the intervention

Three main factors were identified. Case managers have large caseloads that increasingly involve complex clients. This, coupled with the limited amount of time available, makes it difficult to sit down with a client and to take the time that is needed for the intervention. One of the professionals explained:

Often it’s really a matter of investing, of time… And that, from day-to-day you just lack time. That is what’s lacking. That you want to take the time, but that you still have to go like “oh, in half an hour I have to go to the next appointment.” So there is always pressure there, like, I have to… And my clients notice that too (P10).

The professionals argued that it was difficult to implement a new intervention and learn new skills because they had busy schedules, especially when other matters were more pressing (e.g., client crises). This issue was compounded by the high turnover in the participating teams during the 20-months of the study. This resulted in a greater focus on primary tasks and making sure clients were taken care of. The teams’ knowledge and understanding of the intervention were correspondingly impacted, and new case managers had to be trained up.

Second, Case managers and experts-by-experience felt that the importance of the intervention was not sufficiently recognized. Neither time nor space was available in their daily schedule, and there was a great deal of misalignment and poor communication. What would have helped, according to the professionals, was: (1) making the intervention part of formal processes, for example in electronic patient systems or in yearly team performance evaluations, and having it regulated and approved by management; and (2) creating the necessary room in people’s schedules where they could apply it:

It must also be supported by management and seen as important. So I think it’s from the bottom up, but also um… from the organization that’s behind it. You can’t expect people to have to do all sorts of things and then also have to implement… something themselves (P15).

The professionals indicated that, as case managers tend to work individually, it would have helped to integrate the intervention into the team setting, for example, in regular crisis meetings. Implementation would have also benefited from having a designated individual in each team who secured its place on the agenda. Additionally, some suggested that yearly booster training sessions would be helpful.

### Perceived added value of the intervention

According to the professionals, the main added value of the intervention was that it forced them to sit back, listen, and take their time. This helped to create insight (for both themselves and their clients) and openings to discuss victimization experiences. As clients will usually only talk about negative experiences when the professional explicitly discusses them, the intervention made discussing what is a heavy and difficult topic more straightforward.

Because it’s always about vulnerable things. Things that people would rather not… That would rather not be there. And the more you have of those kinds of things the more you actually forget that they play a role. And sometimes it is also just eh for the client very enlightening to eh to get the insight. Why do I do what I do? What exactly is there then… Why do I always fall into the same trap or why do I never succeed or… What is it exactly? As far as I am concerned, this is a very nice instrument (P12).

What was also achieved was that “you are connecting (with the client), you are giving someone the idea of ‘hey, he/she is interested in what happened, why I am the way I am.”’ (P13).

The intervention provides a structured way of addressing the issue of caseloads and explicitly paying attention to clients who seem to be doing fine or are mentally stable but are not making much progress on societal participation. By using the intervention on a regular basis (and not only at intake), the professionals stated that they had gained insights into why their clients were struggling with societal participation or avoiding it altogether, what influenced certain choices regarding same, and learning about the clients’ wishes regarding rehabilitation.

The responses of the clients to the intervention, according to the professionals, were generally positive. However, at first, some clients resisted discussing victimization experiences because they were distressing. Returning to them in a subsequent appointment seemed to help resolve this sticking point. Professionals who initiated the Victoria conversation by starting with negative experiences rather than with participation struggles noticed that clients were reluctant to take part. On the other hand, the professionals who started by trying to connect with the client, took their time, acknowledged feelings associated with negative experiences, and related them to struggles with participation, received positive feedback.

The professionals said that the intervention influenced their daily practice. The most important effect was that their overall awareness of both victimization and participation increased. Several professionals acknowledged that beforehand, they were less conscious of the impact of victimization experiences, but subsequently, they noticed avoidance or stagnation with participation more. Additionally, they mentioned that they could no longer simply assume that their clients were satisfied with their social functioning, but checking-in frequently and “connecting with the client, and not so much having a biased, but an inquisitive attitude. Well, that works” (P4).

### Clients’ conversations with professionals

Most of the clients discussed victimization or other setbacks related to societal participation felt that it was important to have such conversations (Table 5), though they did not think they had to discuss these topics more often.

**TABLE 5:** Clients on social activities and victimization or setbacks at 10- and 20-month follow-up (*N* = 326 at T1; *N* = 315 at T2).

	T1		T2	
	Intervention *M* (SD)	Control *M* (SD)	Intervention *M* (SD)	Control *M* (SD)
I have talked to my case manager in the FACT team about these kinds of experiences.	2.78 (0.99)	2.93 (0.92)	3.01 (0.70)	2.86 (0.89)
I don’t talk enough about these kinds of experiences with my case manager.	1.64 (1.08)	1.50 (1.16)	1.65 (1.05)	1.63 (1.06)
I think it is important to talk about this experience with my case manager.	2.98 (0.87)	2.99 (0.81)	2.98 (0.77)	2.88 (0.87)
I find talking to my case manager about setbacks enlightening.	2.87 (0.92)	3.03 (0.71)	2.89 (0.77)	2.70 (0.90)
After discussing these experiences with my case manager, I feel relieved.	2.80 (0.88)	2.91 (0.79)	2.78 (0.77)	2.70 (0.79)
Talking to my case manager makes me feel that I have been heard.	2.96 (0.78)	2.93 (0.82)	2.91 (0.82)	2.84 (0.87)
Talking to my case manager makes me feel less uncomfortable.	2.67 (0.95)	2.82 (0.82)	2.68 (0.80)	2.59 (0.89)
Talking to my case manager will ensure that I am better able to deal with these kinds of situations in the future.	2.79 (0.83)	2.73 (0.87)	2.63 (0.81)	2.61 (0.90)
Talking about such experiences with my case manager helps in my recovery process.	2.86 (0.84)	2.85 (0.85)	2.80 (0.80)	2.71 (0.90)

Minimum = 0, maximum = 4.

Many of the clients mentioned in their interviews that they had issues with participation (such as a lack of familial contact, a small social network, and wanting voluntary work); only a few mentioned discrimination or victimization (such as arguments with neighbors and traumatic encounters with the police). Even though victimization was rarely a topic of conversation with their case manager, many clients discussed it with their family, social contacts, and in relation to their needs regarding societal participation. Additionally, many of their conversations were about daily matters, such as how the week had been going, or medication and symptoms.

In general, the clients do not recognize that a new intervention was being used. They claimed that conversations on victimization were rarely followed up. They received different types of advice from their case manager, but few were rehabilitation-related and were often practical. However, most of the clients were satisfied with their case manager; they felt a connection with them and that they were interested in what they liked doing. As was the case with the structured interviews, what they gained most out of their conversations was that they could vent their feelings, and they felt listened to and supported.

It is a kind of handholding … if things were going a bit less or so, I can always go to her. And we can then, you know, she can always assess how things are going, and yes, that is also the reason that when I e-mailed so to speak. Sometimes I e-mailed because I eh… because I then, yes I did not really feel good, and worried much eh… or something. And often it’s better after I’ve written it off, so to speak, because then I’ve shared it and I know okay, you know, it’s known and that always gave me peace (C16).

## Discussion

The present process evaluation aimed to understand the trial effects by examining the implementation process (including training and use of the intervention), the factors that influenced this process, and the impact the intervention had on the clients. The results show that the professionals shared the urgency of paying attention to victimization and discrimination and its harmful effects on societal participation. They also found the intervention steps to be logical and the intervention protocol easy to use. Even though they said they discussed victimization more often with their clients, they did not always follow the steps. Furthermore, they remained reluctant to initiate conversations about victimization, mainly due to a fear that their clients might relapse, become traumatized, or feel insecure or uncomfortable about bringing it up and talking about it. However, when the professionals began a conversation on victimization, their subsequent experiences were positive. The clients did not relapse, which confirmed the findings of previous studies (20). Additionally, they felt acknowledged. Using the intervention gave the professionals insights into their clients’ rehabilitation wishes and allowed them the opportunity to discuss victimization experiences.

The different means we used to assess the extent of use of the Victoria intervention showed some discrepancies. The professionals indicated in the checklist that they mostly followed the intervention steps, and similarly, in the structured questionnaires, they reported that they used the intervention on over half of the clients. However, in the interviews and intervision meetings, even though they indicated they were more aware of victimization and addressed the topic more often, they took a flexible approach to the application of the intervention. Furthermore, they stated in the interviews that they were reluctant to use it and did so only cursorily.

The findings indicate that the professionals need more training in how to address victimization and should sit on their hands more often when engaged in conversations on this difficult topic. Extending the current intervention with more in-depth and real-life role-plays using actors and peer workers is advised, as well as annual refreshers.

What was notable was that the RCT showed the positive effects of the intervention on the discrimination that the clients experienced and on the acknowledgment of this (16). It seemed that just addressing victimization helped societal participation. Higher fidelity to and the stricter use of the Victoria intervention might have enlarged the positive effects and improved other outcomes (including societal participation and victimization).

Several factors associated with victimization are often examined, including ethnicity. However, evidence is inconclusive; some studies found that ethnicity was a risk factor (35), and others established it as a (partially) protecting factor; when the majority in a (bad) neighborhood has the same ethnicity as the client, this appears to be protective (36). On the contrary, Dutch studies do not show a difference between victims and non-victims in relation to ethnicity (37, 38). In our effect study (16), we also found that it had no predictive value on the outcome measures. As it may still have an influence on the dynamic of the conversations on victimization between mental health staff and their clients, and this was not addressed in the semi-structured interviews, future studies should address this topic more directly.

Mental healthcare still focuses on the treatment of symptoms rather than rehabilitation (39). Concentrating on victimization experiences may lead to an overemphasis on the pain and emotions that clients experience. Several of the professionals used victimization experiences as the starting point of the intervention rather than participation issues, and this presented the possibility of the aforesaid. This accords with previous research (40). Traumatic experiences should not be dwelt upon, but they should be treated. Such an approach is incorporated in the Victoria intervention in several respects. Those implementing it are required to start by discussing struggles with participation. In the third step, the mindset shifts away from the victimization experience toward the wish to participate. Finally in the fourth step, one of the options is trauma-focused treatment. Even though we paid attention to the need not to dwell on recent victimization experiences, future implementation of the intervention (and the accompanying training) should concentrate on this more.

In the training, intervision sessions, and interviews, we found that experts-by-experience understood the essence of the intervention and that it required time, but questioned whether they had the time for it. They pointed out that they were already using large parts of it. These findings were in keeping with previous studies. First, experts-by-experience adopted a strengths-based approach more than the other professionals (41), which can empower and instill hope in clients. By disclosing their own stories, it is possible for them to regain control over their illness (41, 42). Second, because the nature of the contact in the intervention is more low-key than is usual, so is the distance between the professional and the client. Third, experts-by-experience can discuss day-to-day life, such as how to overcome stigma, more often than would be the case with, say, psychologists (42). This also encourages empathy, because experts-by-experience have similar experiences and can relate more to their clients (43). In future implementations of the intervention, case managers will have to undergo a greater attitude shift than experts-by-experience.

We discovered that there were significant barriers to the use of the intervention on a regular basis, for example, increasingly heavy caseloads. This was a function of the way FACT teams tend to work. The professionals stated that 80% of their days should be spent on clients, but they have busy schedules and so have only a limited amount of time. The role of management in successful implementation is well-known (44); we also found that a lack of support resulted in diminished use of the intervention. This needs to be addressed. The intervention should be made part of treatment plans and a component of team performance indicators.

Our intervention was interwoven into the Boston Psychiatric Rehabilitation method (BPR) (31). All the teams involved in the intervention were certified FACT teams; however, the literature shows that scores on rehabilitation items are lower than other scores that comprise the overall FACT fidelity score (45). Our findings confirmed this; while all the teams were trained in the BPR, adherence to rehabilitation principles was insufficient. We learned in the interviews that team meetings focused on crises and that the professionals worked mostly individually. A recent study of FACT teams (46) concluded that, although their fidelity scores were low, rehabilitation efforts increased over time. However, when rehabilitation principles had been more standard practice among the teams in the present study, the Victoria intervention would have offered even greater improvements.

In addition, the FACT teams’ mission was ambiguous. This is an important issue because mission determines successful implementation. In Netherlands, FACT teams provide care for relatively stable clients using rehabilitation methods and support them during psychiatric crises. Continuity of care provides stability for clients. However, due to recent policy changes (18), FACT teams have been forced to adopt a more treatment-oriented approach and focus less on care *per se*. Together with regional differences in client populations and organizational networks, FACT teams specialize in, for example, first psychosis episodes or addiction, or focus on treatment at the expense of rehabilitation. The Victoria intervention can help address this issue.

### Strengths and limitations

The present study has yielded valuable information on the significance of recognizing and addressing victimization in clients and the difficulties professionals have discussing victimization with their clients. Other strengths of the study include its mixed-methods design and the large sample of participants and number of observations. The study has some limitations. First, we included clients who received the intervention according to their case managers, so we could not measure its true extent. Secondly, while we measured the extent of use by employing several methods, we did not design a bespoke fidelity scale that might have proven more accurate.

## Conclusion

Results from this process evaluation indicate that the intervention increased awareness of victimization. Even though the steps of the intervention were not always followed as they should have, the professionals involved reported positive experiences. The intervention gave them greater insights into their clients’ rehabilitation wishes and allowed them to discuss victimization experiences. This process evaluation has an important added value in that it gave us a better understanding of the effect evaluation of the intervention. Our findings might facilitate the development and implementation of other interventions in community mental healthcare settings. In particular, it is hoped that the Victoria intervention might aid clients’ societal participation.

## Data availability statement

The datasets presented in this article are not readily available because the data that are used in this study contain sensitive patient information, such as their diagnosis and information about criminal victimization. For this reason, there are restrictions on public data sharing. The funding body, NWO, imposed a contract with the data repository Data Archiving and Networked Services (DANS), to encourage long-term storage and availability for further research. After the entire study is finished, DANS will make the data available upon reasonable request via https://www.narcis.nl/. Requests to access the datasets should be directed to WA, w.m.m.albers@tilburguniversity.edu..

## Ethics statement

The studies involving human participants were reviewed and approved by the Medical Ethical Committee of the Elisabeth Hospital in Tilburg (NL53845.028.15) on November 18, 2015, for all participating sites. The study was registered with the Dutch Trial Register (NL4172). The patients/participants provided their written informed consent to participate in this study.

## Author contributions

WA recruited participants and research assistants conducted the interviews. WA, YN, and DR carried out the analyses. All authors were involved in the study’s conceptualization and design, contributed to the article, and approved the submitted version.
