# Supplementary material for: Addressing victimization to enable societal participation in flexible assertive community treatment: A process evaluation of the implementation of a new intervention
Source: Front Psychiatry. 2022 Sep 20;13:956133. doi: 10.3389/fpsyt.2022.956133 (PMC9530599; doi:10.3389/fpsyt.2022.956133)
Supplement: Supplementary file 1 [file Data_Sheet_1.docx]

Supplementary Material

**Table S1. Rehabilitation principles in treatment plans (*N* = 125)**

| ***Plan level*** | ***n*** | **%** |
| --- | --- | --- |
| Rehabilitation goal formulated | 122 | 97.6 |
| Signing space for service user consent | 104 | 83.2 |
| Date planned for treatment plan evaluation | 84 | 67.2 |
| Plan formulated in the first person | 75 | 60 |
| Emergency agreements | 51 | 40.8 |
|  |  |  |
| ***Goal area present in treatment plan*** |  |  |
| Daytime activities | 24 | 19.2 |
| Social contacts | 58 | 46.4 |
| Housing | 56 | 22.8 |
| Work | 56 | 44.8 |
| Learning | 5 | 4 |
| Meaning in life | 48 | 38.4 |
| Self-care | 62 | 49 |
| Mental health | 113 | 90.4 |
| Physical health | 39 | 31.2 |
|  |  |  |
| ***Degree of adherance to rehabilitation principles - Quality score*** |  |  |
| 1 (0-3) No adherence | 22 | 17.6 |
| 2 (4-6) Insufficient adherence | 90 | 72 |
| 3 (7-8) Sufficient adherence | 13 | 10.4 |
| 4 (9-10) Substantial/full adherence | 0 | 0 |

**Table S2. Case managers and experts-by-experience on victimization and Victoria conversations on 10- and 20-month follow-up *(N = 305 on T1; N = 288 on T2)***

|  |  | **T1** |  | **T2** |  |
| --- | --- | --- | --- | --- | --- |
|  |  | **Intervention  *n* (%)** | **Control  *n* (%)** | **Intervention *n* (%)** | **Control  *n* (%)** |
| Does this client avoid social participation? | No, not at all | 30 (19%) | 47 (32%) | 42 (27.8%) | 48 (35%) |
|  | Not really | 59 (37.3%) | 41 (27.9%) | 40 (26.5%) | 39 (28.5%) |
|  | Yes, somewhat | 49 (31%) | 37 (25.2%) | 52 (34.4%) | 34 (24.8%) |
|  | Yes, definitely | 20 (12.7%) | 18 (12.2%) | 17 (11.3%) | 16 (11.7%) |
|  | Not applicable | 0 (0%) | 4 (2.7%) | 0 (0%) | 0 (0%) |
| Has this client stagnated in or stopped social activities? | No, not at all | 22 (13.9%) | 40 (27.2%) | 32 (21.5%) | 36 (26.3%) |
|  | Not really | 64 (40.5%) | 45 (30.6%) | 47 (31.5%) | 50 (36.5%) |
|  | Yes, somewhat | 55 (34.8%) | 33 (22.4%) | 50 (33.6%) | 33 (24.1%) |
|  | Yes, definitely | 13 (8.2%) | 23 (15.6%) | 18 (12.1%) | 18 (13.1%) |
|  | Not applicable | 4 (2.5%) | 6 (4.1%) | 2 (1.3%) | 0 (0%) |
| Is this due to victimization? | Yes | 61 (63.5%) | 31 (37.8%) | 51 (56%) | 29 (37.7%) |
|  | No | 31 (32.3%) | 45 (54.9%) | 37 (40.7%) | 41 (53.2%) |
|  | Don't know | 4 (4.2%) | 6 (7.3%) | 3 (3.3%) | 7 (9.1%) |
| In the past 9 months, have you ever had conversations about this victimization or other setbacks? | Yes | 130 (82.8%) | 126 (85.7%) | 120 (79.5%) | 99 (73.3%) |
| If yes, how often? | (Almost) never | 15 (11.5%) | 8 (6.3%) | 12 (10%) | 15 (15.2%) |
|  | Rarely | 18 (13.8%) | 18 (14.3%) | 14 (11.7%) | 12 (12.1%) |
|  | Occasionally | 66 (50.8%) | 74 (58.7%) | 75 (62.5%) | 58 (58.6%) |
|  | Often | 31 (23.8%) | 26 (20.6%) | 19 (15.8%) | 14 (14.1%) |
| Have you held a 'Victoria' conversation with this client in the past 9 months? | Yes | 83 (55%) | - | 87 (60%) | - |
| If yes, how often? | (Almost) never | 15 (18.3%) | - | 17 (21.3%) | - |
|  | Rarely | 12 (14.6%) | - | 16 (20%) | - |
|  | Occasionally | 43 (52.4%) | - | 38 (47.5%) | - |
|  | Often | 12 (14.6%) | - | 9 (11.3%) | - |
| Did you also discuss the content of the Victoria conversation with your colleagues? | Yes | 40 (29.4%) | - | 57 (43.2%) | - |
